# Complete Recovery From Amniotic Fluid Embolism With Cardiac Point-of-Care Ultrasound (POCUS) and Rotational Thromboelastometry (ROTEM)-Guided Resuscitation During Cesarean Section: A Case Report

**DOI:** 10.7759/cureus.101008

**Published:** 2026-01-07

**Authors:** Alexandra Carneiro, Susana Maia, Beatriz Xavier, Catarina Sampaio, Pilar Miguelez

**Affiliations:** 1 Anesthesiology Department, Unidade Local de Saúde de Trás-os-Montes e Alto Douro, Vila Real, PRT

**Keywords:** amniotic fluid, disseminated intravascular coagulation, embolism, obstetrics, ultrasonography

## Abstract

Amniotic fluid embolism (AFE) is a rare but severe obstetric emergency, with mortality approaching 50% within the first hour. The diagnosis of AFE is clinical and requires a high index of suspicion, along with the consideration of multiple differential diagnoses in peripartum patients presenting with sudden cardiovascular collapse. We report the case of a 38-week pregnant woman who, following cesarean section due to stationary labor, suffered a cardiac arrest during placental extraction. Following the return of spontaneous circulation, she exhibited bilateral decerebrate posturing and a severe coagulopathy consistent with disseminated intravascular coagulation (DIC). Cardiac point-of-care ultrasound (POCUS) revealed right heart chamber dilation, while rotational thromboelastometry (ROTEM) demonstrated profound hemostatic failure, guiding massive transfusion and hemodynamic support. Additional imaging ruled out pulmonary embolism and other differential diagnoses. This case report highlights the value of cardiac ultrasound and ROTEM for the prompt identification and guided resuscitative management of AFE. With multidisciplinary intensive care, the patient achieved complete neurological recovery and was discharged without deficits.

## Introduction

The majority of amniotic fluid embolism (AFE) cases manifest during labor, with approximately 19% occurring during cesarean deliveries (CD) [1]. Its origin remains unclear; it has been suggested that it is an anaphylactic syndrome of pregnancy involving the complement system, causing vasospasm, edema, and early onset disseminated intravascular coagulation (DIC) [2,3]. AFE is an exclusion diagnosis, requiring a high level of suspicion in peripartum patients experiencing sudden cardiovascular collapse or cardiac arrest (87%), with other presentations including respiratory collapse and DIC [1,2]. The associated rapidly changing coagulation profile means that laboratory testing is impractical in informing real-time clinical decision-making [4]. This case report portrays the crucial role of cardiac ultrasound and rotational thromboelastometry (ROTEM) in the early recognition and prompt management of this syndrome.

## Case presentation

A 31-year-old pregnant woman (38+4 weeks) was scheduled for induction of vaginal delivery with labor epidural analgesia. She was classified as physical status American Society of Anesthesiologists (ASA) II and had a history of recurrent spontaneous abortions. The vital signs were normal at the time of hospitalization. All laboratory values are presented with their corresponding RR (reference ranges). Blood analysis showed a mild thrombocytopenia (143,000/mL; RR 150,000-450,000/mL), with normal haemoglobin value (11.4 g/dL; RR 11.0-16.0 g/dL for pregnancy), as well as normal renal and liver function, and coagulation. ECG revealed a sinus rhythm without abnormalities.

A CD was decided due to the stationary labor, and the patient was transferred to the operating room. An epidural top-up bolus was administered using 112.5 mg ropivacaine 0.75%, without complications. After fetal extraction and during placental extraction, the patient experienced sudden coughing followed by asystole. Prompt cardiopulmonary resuscitation (CPR) was initiated with epinephrine 1 mg IV (intravenous), which led to a return of spontaneous circulation after one cycle. However, the patient remained unconscious with bilateral decerebrate posturing. A female newborn with an Apgar score of 9 and 10 (at first and fifth minutes, respectively) was delivered.

**Figure 1:**
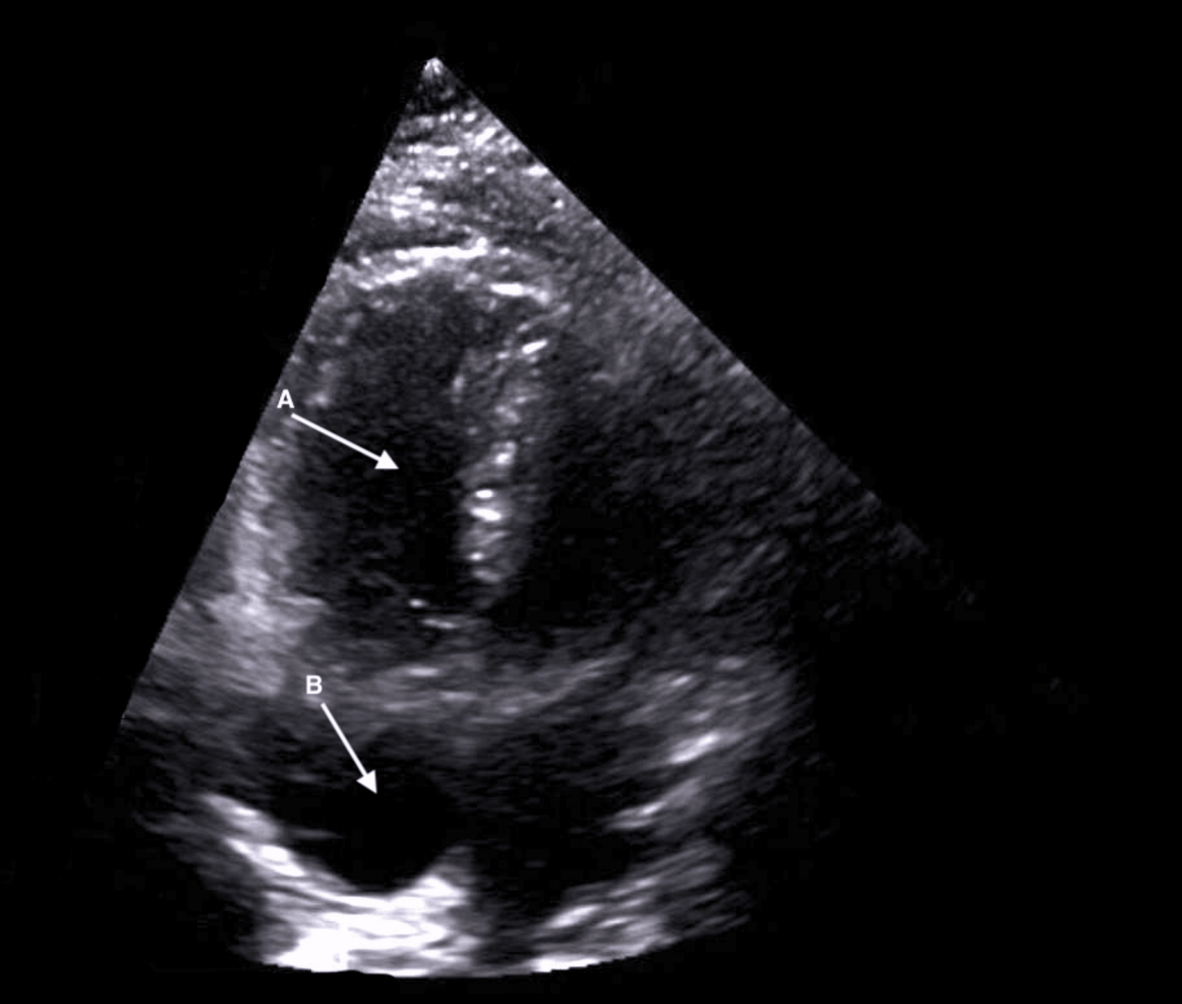
POCUS in the apical four-chamber view performed during resuscitation, showing right ventricular (arrow A) and right atrial (arrow B) dilatation POCUS: point-of-care ultrasound

**Figure 2:**
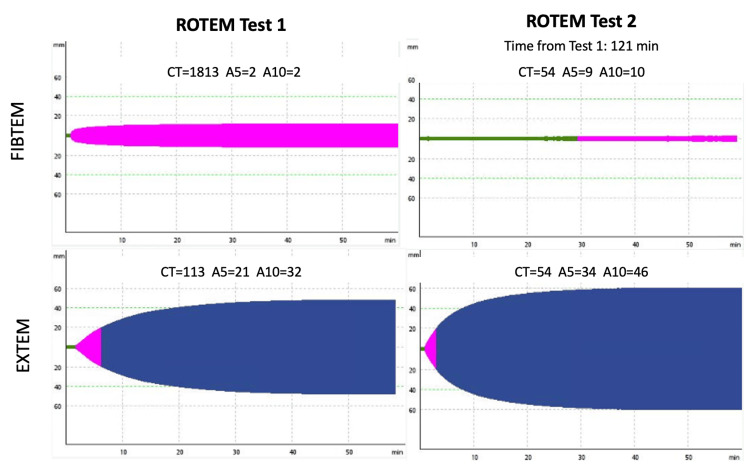
Point-of-care ROTEM tests (FIBTEM and EXTEM) performed during resuscitation Test 1 during cardiac arrest. Test 2 in the Emergency Department. Normal values are FIBTEM A5 ≥ 12 mm and EXTEM clotting time (CT) <75 s. ROTEM: rotational thromboelastometry

After orotracheal intubation, a central venous plus a brachial artery catheter were inserted. Arterial blood gas (ABG) analysis at that moment was pH 7.34 (RR 7.350-7.450), oxygen partial pressure (PaO2) of 18 mmHg (RR 71.0-104.0 mmHg), partial pressure of carbon dioxide (PaCO2) of 48 mmHg (RR 35.0-46.0 mmHg), oxygen saturation of 23% (RR 94.0-98.0%), and lactates of 6.1 mmol/L (RR 0.5-2.0 mmol/L). A cardiac point-of-care ultrasound (POCUS) (Figure 1) showed right ventricle (RV) dilation and right atrial (RA) enlargement with preserved cardiac contractility. Aggressive fluid resuscitation and infusion of norepinephrine were initiated. Laboratory investigations revealed acute anemia (7.6 g/dL) and moderate thrombocytopenia (66 000/mL), prolonged prothrombin time (18.0 seconds, RR 9.0-13.0 seconds), prolonged activated partial thromboplastin time (78.4 seconds; RR 28.0-38.0 seconds), an INR of 1.36 (RR < 1.2), hypofibrinogenemia (183 mg/dL; RR 200.0-400.0 mg/dL), normal liver enzymes, and normal renal function. Despite minimal blood loss, DIC was confirmed, and AFE was suspected. Tranexamic acid (1 g) and two units of packed red blood cells (pRBC) were administered. In addition to laboratory bloods and blood gas samples, a point-of-care ROTEM was performed (Figure 2).

At the end of the procedure, the patient was hemodynamically stable, with only minimal vaginal blood loss observed, and the uterus was adequately contracted. She was therefore moved to the Emergency Department.

**Figure 3:**
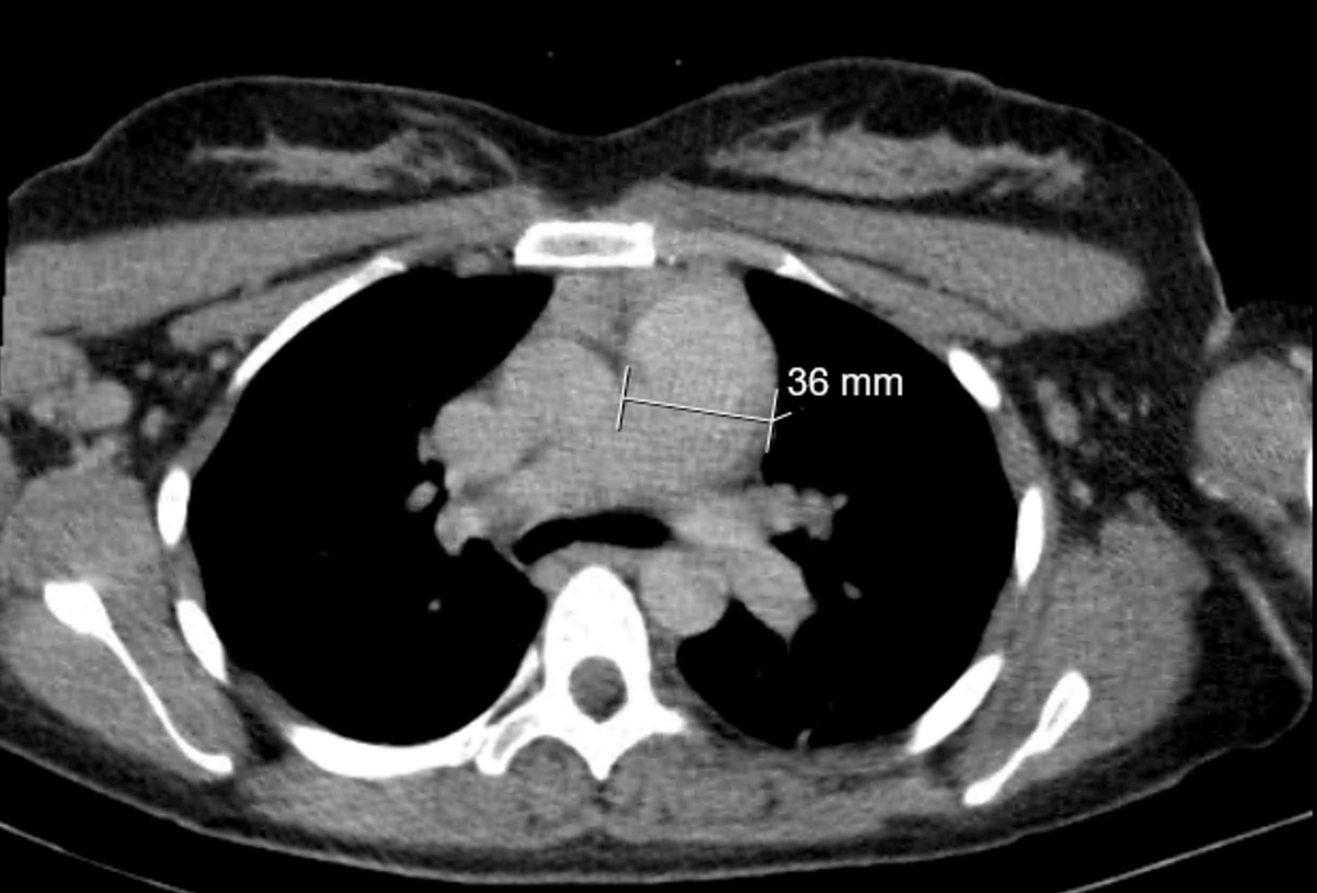
Thoracic CT in the axial plane obtained prior to admission to the intensive care unit

In this unit, the parturient required norepinephrine only transiently immediately after the cardiac arrest, but subsequently showed a tendency toward hypertension, which improved after the initiation of sedation and analgesia. Once stabilized, the patient underwent a thoracic computed tomography (CT) (Figure 3), which revealed an enlarged pulmonary artery trunk measuring 36 mm, with no evidence of pulmonary embolism (PE) or findings suggestive of acute respiratory distress syndrome (ARDS). Ultrasound of the lower limbs excluded deep vein thrombosis. Another ROTEM was performed (Figure 2), and the results prompted the administration of one unit of packed red blood cells (pRBC), three units of platelets, six units of fresh frozen plasma, and four grams of fibrinogen concentrate up to that point.

Initial neurological examination revealed a comatose patient with closed eyes and an initially midline gaze that later became horizontally disconjugated. Oculocephalic reflexes were absent, and pupillary and corneal reflexes were bilaterally indifferent. The patient exhibited bilateral decerebrate posturing and weak deep tendon reflexes. Brain and neck contrast-enhanced CT angiography revealed spontaneous hyperdensity in the cortical sulci of the frontal regions, suggestive of subarachnoid hemorrhage compatible with DIC, with no other abnormalities detected. Neurosurgery found no indication for surgery.

Four hours post-surgery, the patient experienced neurological deterioration, presenting with mydriatic, non-reactive pupils; absence of corneal, oculocephalic, and gag reflexes; and no response to nociceptive stimuli. A repeat brain CT showed no new findings, and the patient was admitted to the Intensive Care Unit (ICU).

On the first postoperative day, a transthoracic echocardiogram showed dilated right heart chambers, a mildly reduced left ventricular ejection fraction (LVEF) of approximately 50%, mild mitral and mild-to-moderate tricuspid regurgitation, and excluded the presence of a patent foramen ovale. These findings were compatible with AFE. The electroencephalogram showed a trace without specific pathological alterations. Brain MRI demonstrated a linear hyperintense signal in the right pontine region, suggestive of hemorrhage, with no other abnormalities observed.

Twenty-four hours post-surgery, the patient was responsive to commands, with no apparent neurological deficits, and was therefore successfully extubated.

During the four days in the ICU, the patient continued to present with anemia, thrombocytopenia, and mild coagulopathy, requiring transfusion of pRBC and platelets, while showing progressive improvement. The patient was admitted to the Neurology ward on the fifth day and was discharged on the eighth day with complete reversion of the neurological deficits. At the six-month follow-up, the patient exhibited no neurological sequelae, and brain MRI revealed complete resorption of subarachnoid hemorrhagic residues, leading to discharge from neurological care.

## Discussion

AFE is a diagnosis of exclusion in pregnant or postpartum women presenting with acute cardiorespiratory compromise, requiring consideration of other potential differential diagnoses[1,2]. The most common obstetric condition sharing features with AFE is hemorrhagic shock [2]. However, in this case, the patient was not anemic prior to delivery and did not experience intrapartum hemorrhage, which helped rule out this condition. Anaphylaxis was also unlikely given the absence of premonitory symptoms such as rash or a period of hypotension before cardiovascular decompensation [2]. High spinal anesthesia and local anesthetic toxicity were also excluded, as DIC is rare in these syndromes, and the patient did not exhibit any typical clinical features [2]. Septic shock was also excluded because it is unlikely to cause sudden cardiovascular collapse as occurred in this case, and the patient did not have a fever or other signs of the classic systemic inflammatory response syndrome (SIRS) [1]. Although both eclampsia and HELLP (Hemolysis, Elevated Liver enzymes, and Low Platelet count) syndrome can present with thrombocytopenia and coagulopathy, there was no elevation of liver enzymes, and the patient did not exhibit hypertension, edema, proteinuria, headache, or seizures prior to the collapse, making these diagnoses unlikely [1]. Therefore, the initial hypotheses considered were AFE and massive PE, given the presence of a premonitory syndrome (coughing), the abrupt onset of multiorgan failure, and the presence of risk factors associated with both conditions in this case. The profound hypoxemic respiratory failure and elevated lactate levels observed on ABG analysis further supported both hypotheses [2]. Although less frequent, DIC may also develop in cases of massive PE or after cardiac arrest, and its presence should not dissuade the clinician from eliminating PE as a possible diagnosis [5]. However, the presence of RV failure and a negative scan for PE could be considered important criteria to diagnose AFE [5].

Cardiac POCUS is a crucial tool for differentiating life-threatening causes of hypotension and cardiac arrest, reducing diagnostic ambiguity in AFE cases, and identifying patients needing advanced hemodynamic support [6]. In this case, it enabled the prompt detection of the classic RV dilatation seen in AFE, supporting the diagnosis. The absence of acute left ventricular failure, regional wall motion abnormalities, pericardial effusion, or valve rupture made the diagnosis of cardiogenic shock less likely [5]. Additionally, DIC is rare in early cardiogenic shock, and the patient had no prior diagnosis of cardiomyopathy or risk factors for ischemic heart disease or arrhythmia, further reducing the likelihood of this diagnosis [2]. ROTEM results showed complete haemostatic failure with a FIBTEM A5 and APTEM clotting time (CT), consistent with a severe consumptive coagulopathy. This coagulopathy was more characteristic of AFE than PE, making AFE the most likely diagnosis, and antifibrinolytic therapy was therefore not initiated [4].

The markedly abnormal ROTEM results also prompted immediate communication between the anesthetic and hematology teams, who rapidly coordinated a patient-tailored transfusion of blood components--an intervention that proved essential during the patient’s resuscitation.

The thoracic CT and lower limb ultrasound performed subsequently were important in definitively excluding PE and confirming that no targeted therapy was required. A transthoracic echocardiogram obtained on the first postoperative day revealed further significant signs of right heart failure, including dilated right heart chambers and tricuspid regurgitation, which supported the diagnosis of AFE [1].

## Conclusions

AFE is a rare but catastrophic obstetric emergency characterized by sudden cardiopulmonary collapse, often accompanied by DIC. Management is primarily resuscitative, emphasizing rapid optimization of oxygenation, hemodynamic stabilization, and prompt correction of coagulopathy.

This case of suspected AFE with severe coagulopathy during cesarean section illustrates the value of ROTEM in expediting coagulation assessment and enabling targeted blood product administration. POCUS played a pivotal role in differentiating amniotic fluid embolism from other etiologies of obstetric shock by narrowing the diagnostic considerations between pulmonary embolism and AFE, thereby guiding and refining targeted cardiovascular resuscitation strategies. Although AFE carries a high maternal mortality and poor outcomes among survivors, with a severe potential for neurological impairment, early resuscitation, effective correction of coagulopathy, and involvement of a multidisciplinary team (MDT) are essential for improving prognosis. In conclusion, this case underscores the importance of cardiac ultrasound and ROTEM in facilitating early diagnosis and urgent clinical intervention in AFE, which were key factors in the patient’s successful recovery.
